# Function and Mechanism of Jasmonic Acid in Plant Responses to Abiotic and Biotic Stresses

**DOI:** 10.3390/ijms22168568

**Published:** 2021-08-09

**Authors:** Yun Wang, Salma Mostafa, Wen Zeng, Biao Jin

**Affiliations:** College of Horticulture and Plant Protection, Yangzhou University, Yangzhou 225009, China; MZ120190969@yzu.edu.cn (Y.W.); dh18033@yzu.edu.cn (S.M.); MX120200764@yzu.edu.cn (W.Z.)

**Keywords:** abiotic stress, biotic stress, crosstalk, defense response, jasmonic acid, plant hormones, signaling pathway

## Abstract

As sessile organisms, plants must tolerate various environmental stresses. Plant hormones play vital roles in plant responses to biotic and abiotic stresses. Among these hormones, jasmonic acid (JA) and its precursors and derivatives (jasmonates, JAs) play important roles in the mediation of plant responses and defenses to biotic and abiotic stresses and have received extensive research attention. Although some reviews of JAs are available, this review focuses on JAs in the regulation of plant stress responses, as well as JA synthesis, metabolism, and signaling pathways. We summarize recent progress in clarifying the functions and mechanisms of JAs in plant responses to abiotic stresses (drought, cold, salt, heat, and heavy metal toxicity) and biotic stresses (pathogen, insect, and herbivore). Meanwhile, the crosstalk of JA with various other plant hormones regulates the balance between plant growth and defense. Therefore, we review the crosstalk of JAs with other phytohormones, including auxin, gibberellic acid, salicylic acid, brassinosteroid, ethylene, and abscisic acid. Finally, we discuss current issues and future opportunities in research into JAs in plant stress responses.

## 1. Introduction

Plants live in the ever-changing natural environment and encounter many factors that are not suitable for growth or even survival [1]. These factors are generally divided into biotic and abiotic stressors. The main biotic stressors include pathogenic bacterial diseases, insects, and herbivores, while drought, saline or alkaline conditions, and extreme temperature are considered abiotic stressors [2,3]. As sessile organisms, plants have evolved sophisticated response mechanisms to resist, mitigate, or recover from these stressors. In this respect, the fundamental and important biological question is how plants perceive stress signals and respond to various adverse environmental conditions. Over the past two decades, plant hormones, which are vital regulators involved in sensing and transmitting various environmental signals and subsequent defense responses, have received intense research attention. Currently, nine major classes of natural plant hormones have been described that underlie numerous reactions to environmental signals, including auxin, cytokinin, gibberellin (GA), abscisic acid (ABA), ethylene (ET), brassinosteroid (BR), jasmonic acid (JA), salicylic acid (SA), and strigolactone [4]. Among these hormones, JA is ubiquitous in higher plant species and therefore has attracted great attention in the field of plant stress response and defense mechanisms [5].

JA and its derivatives, including its methyl ester (MeJA) and its isoleucine conjugate (JA-Ile), are collectively called jasmonates (JAs) [6]. Aromatic MeJA was first isolated from the essential oil of *Jasminum grandiflorum* L. [7]. Free JA was examined and isolated from the culture filtrate of *Lasiodiplodia theobromae*, and was found to inhibit plant growth [8], providing the first report of the physiological function of JA. JAs play a variety of regulatory roles in plant growth and development, for example, axis elongation during embryogenesis, flower development, leaf senescence, root formation, and stomatal opening [9,10]. In addition to growth and development, many studies have shown that JAs improve plant stress tolerance via JA signaling pathways under various adverse environmental conditions.

**Figure 1:**
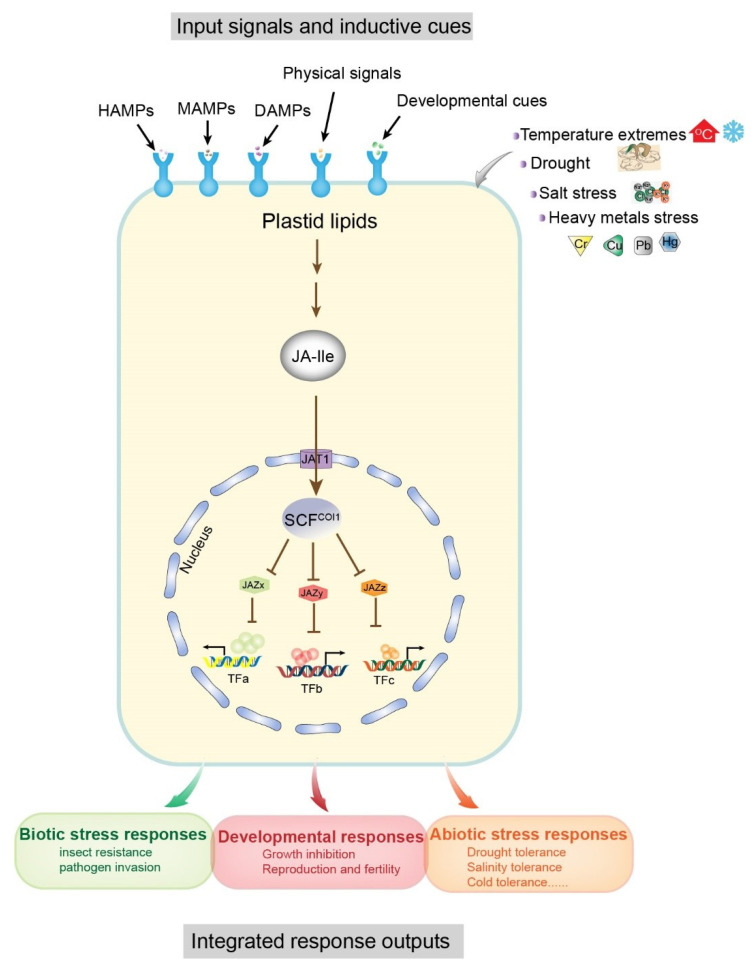
Input signals and output responses of the core jasmonoyl-L-isoleucine (JA-Ile) signaling pathway. Associations of the modular design of JA-Ile signaling with a variety of inductive cues (top) and physiological output responses (bottom) are shown as a schematic diagram. Inductive signals include biotic stresses (microbe and herbivore) and damage related to molecular patterns (MAMPs, microbe-associated; HAMPs, herbivore-associated; and DAMPs, damage-associated) arising from damaged plant cells, attacking organisms, and abiotic stresses. Pattern recognition receptors on the cell surface recognize the signals and resynthesize JA-Ile from plastid lipids. The degradation of multiple JAZ proteins (exemplified by JAZx-z) is promoted by the activation of the E3 ubiquitin ligase SCF^COI1^ and the 26S proteasome. Subsequently, JAZ-interacting transcription factors (exemplified by TFa–c), which govern diverse physiological output responses involved in growth, development, and tolerance to biotic and abiotic stresses, are upregulated due to the degradation of JAZ. The conserved core pathway may be repurposed to connect other input signals and transcription factors. Abbreviations: JA-Ile, jasmonoyl-L-isoleucine; MAMPs, microbe-associated molecular patterns; HAMPs, herbivore-associated molecular patterns; DAMPs, damage-associated molecular patterns; JAZ, jasmonate ZIM domain; SCF, [kinetochore protein 1 (SKP1)-CULLIN1 (CUL1)-F-box]; COI1, coronatine insensitive1; TF, transcription factor. Modified from [12].

Some molecular models of plant–environment interactions include microbe-associated molecular patterns (MAMPs), herbivore-associated molecular patterns (HAMPs), and damage-associated molecular patterns (DAMPs), which are mainly derived from attacking organisms, cell damage, and abiotic stresses (e.g., salt, drought, heavy metals, cold, etc.) (Figure 1) [11,12]. In general, these molecular patterns are associated with JA signaling pathways. The receptor-active conjugated complex JA-Ile is a core component of JA signaling pathways [13,14,15]. As inducing signals, environmental stimuli are recognized by cell surface receptors, triggering de novo synthesis of JA-Ile from plastid lipids, which ultimately results in downstream transcription factor interactions and promotion of growth, development, and specific protective mechanisms in plants [12]. In this reaction, JA-Ile acts to facilitate the interaction between jasmonate zinc-finger inflorescence meristem (JAZ) and coronatine insensitive 1 (COI1) protein within the Skp1p–cullin–F-box protein (SCF) complex, and promotes degradation of JAZ proteins (JAZx, JAZy, and JAZz), resulting in the activation of JAZ-interacting transcription factors (TFs: TFa, TFb, TFc) (Figure 1). These TFs regulate the expression of numerous genes in response to both biotic (e.g., herbivores and fungi) and abiotic (e.g., salt, drought, heavy metals, and cold) stresses and promote specific protection mechanisms (Figure 1) [16,17,18,19]. Therefore, JAs are functionally significant and have attracted extensive research interest in the field of plant–environment interactions.

In this review, we summarize recent progress in JA research, focusing on the biosynthesis and signal transduction of JAs, as well as their roles in biotic and abiotic stress responses.

## 2. Biosynthesis and Metabolism of JAs

### 2.1. JA Biosynthesis

**Figure 2:**
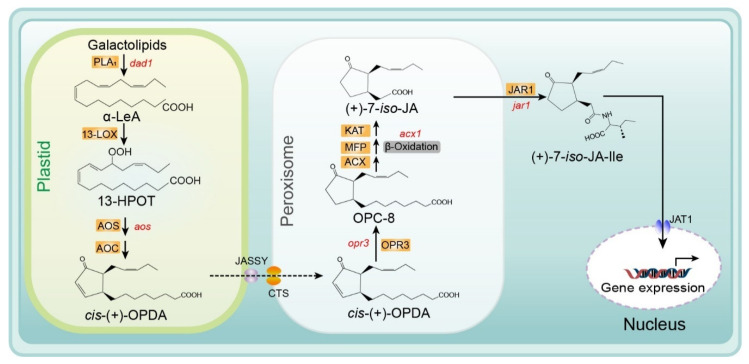
Biosynthesis of JA/JA-Ile initiated by the release of α-linolenic acid from galactolipid. De novo synthesis of JA occurs in the plastids, peroxisomes, and cytoplasm, and JA-Ile is then transported to the nucleus for gene expression. Major genes are indicated in red and enzyme names are highlighted in orange. Abbreviations for compounds: α-LeA, α-linolenic acid; 13-HPOT, (13S)-hydroperoxyoctadecatrienoic acid; cis- (+)-OPDA, cis- (+)-12-oxophytodienoic acid; OPC-8, 3-oxo-2- (2-pentenyl)-cyclopentane-1-octanoic acid; (+)-7-iso-JA, jasmonic acid; (+)-7-iso-JA-Ile, jasmonic acid isoleucine conjugate. Abbreviations for enzymes/proteins: PLA1, phospholipase A1; 13-LOX, 13-lipoxygenase; AOS, allene oxide synthase; AOC, allene oxide cyclase; OPR3, OPDA reductase3; ACX, acyl-CoA oxidase; MFP, multifunctional protein; KAT, 3-ketoacyl-CoA thiolase; JAR1, JA-amino acid synthetase; JASSY, a chloroplast outer membrane protein; CTS, an ABC transporter of the peroxisomal membrane; JAT1, jasmonate transporter 1 (Redrawn based on [30]).

Several reviews of the JA biosynthesis pathway have been published previously. These reviews provide important information on the biosynthesis reactions, genes, and enzymes in this pathway, including explanations of the mechanisms determining enzymatic crystal structure and substrate specificity, and ultimately describe the regulation of JA biosynthesis [20,21,22,23]. Recently, several membrane transporters, including JASSY, comatose (CTS), and jasmonate transporter 1 (JAT1), were reported to function in the JA biosynthesis pathway. In Figure 2, we provide an updated summary of the JA synthesis process, including the reaction steps and names of enzymes and substrates.

The substrate for the biosynthesis of JA is α-linolenic acid (18:3) (α-LeA), which is released from the *sn1* position of galactolipids on chloroplast membranes through the action of phospholipase A1 (PLA_1_) [24]. Next, the substrate is converted to (9S,13S)-12-oxo-phytodienoic acid (OPDA) through the actions of 13-lipoxygenase (LOX), allene oxide synthase (AOS), and allene oxide cyclase (AOC), respectively. All enzymes and products involved in this pathway are located in plastids (chloroplasts). Subsequently, OPDA is transferred from the chloroplast to the peroxisome and transformed into 3-oxo-2- (cis-2ʹ-pentenyl)-cyclopentane-1-octanoic acid (OPC-8:0) by OPDA reductase 3 (OPR3) [6,25,26]. The mechanism of OPDA transport from chloroplasts to the peroxisome remains unclear. Guan et al. identified a protein located on the chloroplast outer envelope membranes that is responsible for exporting OPDA and named it JASSY [27]. For the recipient, CTS—an ABC transporter on the peroxisomal membrane—is the main mediator of OPDA import into peroxisomes [28]. Subsequently, OPC-8 is transformed into (+)-7-*iso*-JA through three β-oxidation reactions by three different enzymes: acyl-CoA oxidase (ACX), multifunctional protein (MEP), and L-3-ketoacyl CoA thiolase (KAT) (Figure 2.) [9,29]. Next, (+)-7-*iso*-JA is transported to the cytoplasm, where it is conjugated with isoleucine (Ile) to form (+)-7-*iso*-JA-Ile—which is considered the most bioactive JA compound—under the action of JAR1 (a JA-Ile synthesizing enzyme). Finally, JA-Ile is transported into the nucleus by the ABC transporter JAT1, where it participates in the subsequent steps of the JA signaling pathway [15] (Figure 2).

### 2.2. JA Metabolism

**Figure 3:**
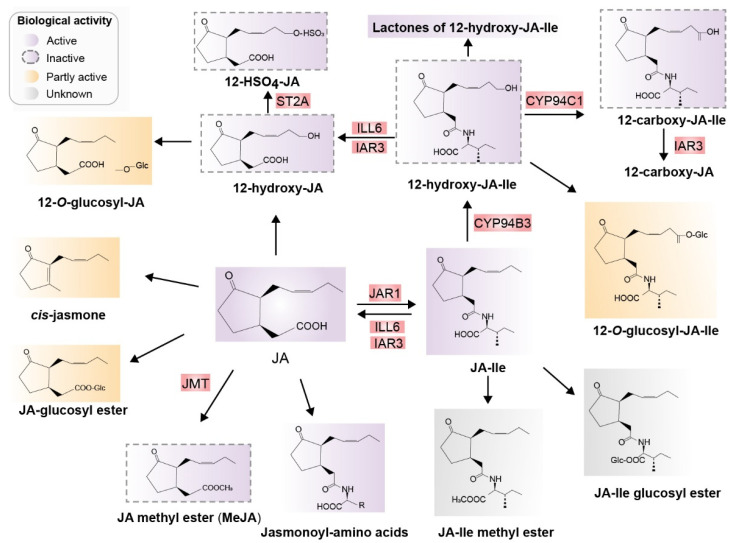
Major jasmonates arising from the metabolic conversion of JA. JA can be methylated to form JA-Me (MeJA) or JA glucosyl ester, decarboxylated to cis-jasmone, or hydroxylated to 12-OH-JA. 12-OH-JA can be sulfated and O-glucosylated, and conjugated with amino acids, preferring isoleucine, which results in JA-Ile. JA-Ile is methylated to JA-Me-Ile. Moreover, 12-hydroxylation of JA-Ile, carboxylation of 12-OH-JA-Ile, O-glucosylation of JA-Ile, and JA-Ile glucosyl ester formation are also possible. Known enzymes involved in these transformations are highlighted in red. Biologically active compounds are in purple and inactive compounds are indicated with dotted outlines in purple. Partially active compounds are shown in yellow, and unknown compounds in gray. Abbreviations: JA, jasmonic acid; JA-Ile, jasmonyl isoleucine; JMT, JA methyltransferase; JAR1, jasmonoyl isoleucine synthetase; CYP94B3, JA-Ile-12-hydroxylase; CYP94C1, 12-OH-JA-Ile carboxylase; the amidohydrolases IAR3 and ILL6; JMT, JA methyltransferase; ST2A, 12-OH-JA sulfotransferase (Redrawn based on [30]).

After biosynthesis of JA and JA-Ile, JA derivatives including active, inactive, and partially active compounds are derived mainly from JA and JA-Ile through at least 12 metabolic pathways. These metabolic pathways include conjugation with amino acids, decarboxylation, sulfation, hydroxylation, carboxylation, o-glycosylation, methylation, esterification, and lactone formation. Consequently, diverse JA derivatives are formed (Figure 3) [30,31].

## 3. JA Signaling

**Figure 4:**
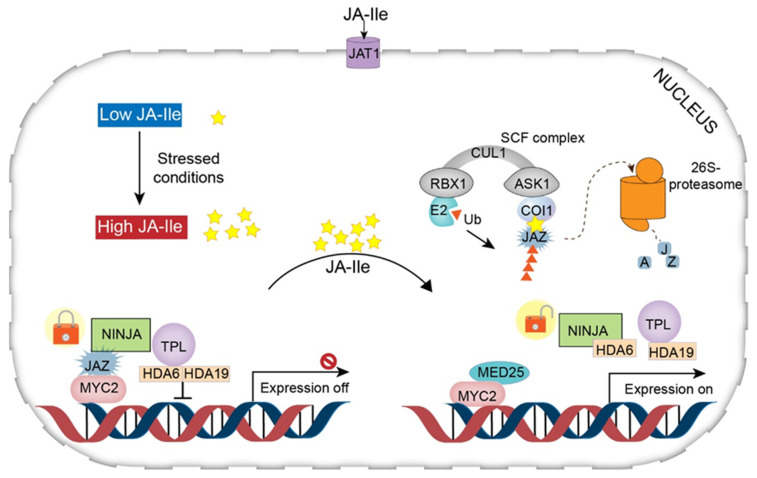
Working model of jasmonic acid signal transduction. Under normal conditions (without stimuli from abiotic stress), with a low level of JA-Ile, activation of the promoters of jasmonate-responsive genes is prevented, as the transcription factors are repressed by JAZ proteins. In this process, TPL and the adaptor protein NINJA are recruited to form an active transcriptional repression complex. Moreover, HDA6 and HDA19 are recruited to change the open complex to a closed one, thereby inhibiting JA responses. Under stress conditions, JA-Ile, which is epimerized from JA, shifts from low to high abundance, and is transported by JAT1 into the nucleus. Then, JA-Ile utilizes the interaction of JAZ with the F-box protein COI1 within the SCF complex, resulting in proteasomal degradation of JAZ. TFs are induced to bind to the G-box element, whereupon MED25 is recruited, activating the expression of jasmonate-responsive genes. Abbreviations: JA, jasmonic acid; JA-Ile, jasmonyl isoleucine; JAT1, jasmonic acid transfer protein 1; JAZ, jasmonate ZIM domain; NINJA, novel interactor of JAZ; TPL, topless; HDA6, HDA19, histone deacetylase 6, 19; Ub, ubiquitin; E2, ubiquitin-conjugating enzymes; RBX1, ring box 1; CUL1, cullin 1; ASK1, *Arabidopsis* SKP1 homolog 1; COI1, coronatine insensitive 1; MED25, mediator 25. (Redrawn based on [19]).

The pathway of JA signaling includes two modes: repression (normal conditions) and activation (stressed conditions) (Figure 4).

Under the repression (normal) condition, the level of JA-Ile in the cytoplasm is very low, and therefore genes involved in JA synthesis remain in the inactivated state [19]. In this state, the gene promoters bind to various TFs that are suppressed by a series of transcriptional repressors called JAZ proteins [characterized by the jasmonate zinc-finger inflorescence meristem (ZIM) domain] [32]. JAZ proteins recruit the protein topless (TPL) and the adaptor protein of JAZ (NINJA) to form an effective closed-complex of JAZ transcriptional repressors (JAZ–NINJA–TPL complex) [33]. This closed complex of JAZ repressors is maintained by further recruitment of histone deacetylase 6 (HDA6) and histone deacetylase 19 (HDA19). This complex can prevent the activation of jasmonate-responsive genes [19,23,30,34].

Under stress conditions, JA-Ile accumulates at high levels in the cytosol and is transferred to the nucleus across the plasma and nuclear membrane through the action of JA transfer proteins (AtJAT1/AtABCG16) [19]. This step is considered the beginning of the JA signaling pathway. In the nucleus, the SCF complex [kinetochore protein 1 (SKP1)-cullin 1 (CUL1)-F-box] mediates JA responses and serves as an E3 ubiquitin ligase. First, JA-Ile transported to the nucleus is recognized by the F-box protein COI1 within the SCF complex. The COI1–JAZ co-receptor complex perceives JA-Ile, promoting the interaction of JAZ with COI1 [35,36]. This interaction leads to the degradation (ubiquitination) of JAZs in the 26S proteasome (Figure 3). Consequently, the lack of JAZ protein (repressor degradation) results in transcriptional activation of TFs and activation of JA-responsive gene expression [16,30]. In addition, mediator 25 (MED25), also designated phytochrome and flowering time 1 (PFT1), a subunit of the *Arabidopsis* mediator complex, connects the TF with RNA polymerase II, modulating gene transcription [19,37,38] (Figure 4). 

Another interesting research area related to JA is the mechanism through which JA signaling is transmitted over long distances to various parts of the plant. When plants suffer from insect herbivory, necrotrophic pathogens, or mechanical wounding, JA and JA-Ile levels increase from the local injury site to distal healthy tissues and undamaged tissues, giving rise to a whole-plant broad-spectrum immunity against secondary challenges [39]. Generally, JA transmission can occur systematically through vascular bundles or the air [40,41]. To date, local wounding of plant tissues has been proposed to alert distal tissues via two mobile signal types (JA-independent and JA-dependent) within the plant. JA-independent signal transmission is represented by three glutamate receptor-like (GLR3) proteins, which are mediated by electrical signals. GLR3 can be quickly transported from leaf to leaf in *Arabidopsis* after leaf injury, resulting in JA burst in distal leaves [42]. Another mobile signal is JA-dependent signal transmission via specific JA molecules [43]. JA can act as a leaf-to-leaf mobile signal driven by two plasma membrane-anchored JA-specific importers (ATJAT3 and ATJAT4, expressed in phloem cells) in damaged *Arabidopsis* leaves, and these two importers mediate intercellular transport of JA along the phloem pathway [39]. In addition, ATJAT1 may be involved in long-distance JA transport [15]. Previous research demonstrated that OPDA and its derivatives, but not the biologically active form of JA-Ile, could be transferred from wounded shoots to healthy roots based on grafting experiments and hormone profiling in *Arabidopsis*. JA can be synthesized and transformed into JA-Ile during its transfer to the root, thereby activating the JA signal transduction pathway [44]. Among individuals, abundant research has revealed that airborne transmission can support the long-distance transmission of a danger signal to initiate plant defenses [6]. JA cannot easily penetrate the cell membrane without carrier assistance, but MeJA can do so due to its high volatility. In recent years, it has been confirmed that MeJA can be transmitted via the air from wounded to healthy leaves or from wounded plants to adjacent plants [44,45].

## 4. JA Hormonal Crosstalk Influences Plant Defense and Development

Plant hormonal signaling pathways control plant growth, development, and environmental responses, and these processes require complex hormonal crosstalk [46,47]. This hormonal crosstalk is intriguingly complex and is often dose-, species-, tissue-, and inducer-specific [48]. Without exception, the JA signaling pathway acts as a major stress hormone pathway that regularly interacts with other plant hormones, creating a complex signaling network linked to other signaling pathways [18,48,49,50]. An increasing number of shared components between JA and various other plant hormone signaling pathways have been identified in recent years. In this section, we introduce the hormonal crosstalk of JA with auxin, GA (gibberellic acid), SA (salicylic acid), BR (brassinosteroid), ET (ethylene), and ABA (abscisic acid) that modulates environmental responses.

### 4.1. JA–Auxin Crosstalk

Auxins are an important hormonal group that plays a crucial role in the growth and development of plants. Various developmental processes in plants are influenced by JA–auxin interactions, including seed development and germination, root growth, flower development, seedling development, tuber formation, and senescence, as reviewed by Xu et al. [51]. Under abiotic and biotic conditions, many physiological processes in plants are controlled by the interaction between indole-3-acetic-acid (IAA)—a type of auxin—and JA, including stem cell elongation, tendril coiling, and production of secondary metabolites [52]. In *Arabidopsis*, auxin can induce the expression of a JA-related gene (*JAZ1/TIFY10A*) that is controlled by the action of the AUX/IAA response transcription factor signaling pathway [53]. JA inhibits root growth and reduces meristem activity [54], and the interaction between JA and auxin mediates root growth inhibition [55]. The interaction between JA and auxin is regulated by MYC2, which binds to the promoters of the auxin-responsive gene *PLT* (plethora, responsible for stem cell niche maintenance and cell division), leading to suppression of its expression and thereby inhibiting root meristem activity [55]. Similarly, in rice plants, root system development is influenced by this interaction after exposure to soil pollutants [56]. Moreover, ERF109 (JA-responsive ethylene response factor 109) mediates JA–auxin crosstalk to regulate lateral root development in *Arabidopsis* [57].

The JA–IAA interaction can also control the development of floral organs. Auxin response factors 6 and 8 (ARF6 and ARF8) are involved in the regulation of floral organ development, including petal expansion, anther dehiscence, stamen filament elongation, cell elongation, and nectary maturation [58]. Both ARF6 and ARF8 stimulate JA production by controlling the JA-responsive TFs MYB21 and MYB24 to promote the growth of floral organs [59].

Leaf senescence is also influenced by JA–auxin crosstalk. Auxin functions as a repressor of leaf senescence—a process induced by JA—resulting in symptoms of lower chlorophyll content, severe yellowing of leaves, and higher cell death rates [60]. Furthermore, exogenous auxin can prevent leaf senescence, which is offset by exogenous JA. In this respect, JAZ4, JAZ8, and IAA29 were reported to be involved in the JA–auxin signaling pathway as negative regulators [61]. The function of JAZs is suppression of the JA signaling pathway [62], and WRKY57 functions as a node of convergence, negatively regulating JA-induced leaf senescence after competitive interactions with JAZ4, JAZ8, and IAA29 [61].

### 4.2. JA–GA Crosstalk

Gibberellins (GAs) are involved in many vital processes in plants such as seed germination, cell division, root and flower development, and fruit set [63]. They also respond to various environmental conditions and inhibit plant growth under stress [64]. Several studies have revealed relationships between GAs and JA under both normal and stress conditions. 

The accumulation of GA under normal conditions causes degradation of DELLA proteins. Under cold stress, GA signaling is reduced, causing plant growth inhibition, while DELLA proteins accumulate [65]. DELLA proteins can bind to JAZ proteins, leading to the activation of jasmonate-responsive genes. This finding illustrates JA–GA crosstalk under stress conditions [66]. JA–GA antagonistic crosstalk has been examined in *Camellia sinensis* during herbivore attack. The results demonstrated that JA–GA signaling regulates the activity of the defensive proteins polyphenol oxidases (PPOs). Elevated JA levels due to the activation of CsPPO2 and CsPPO4 induce PPO activity [59].

In *Oryza sativa*, *OsJAZ9* positively regulates GA and negatively affects JA. *OsJAZ9* interacts with slender rice 1 (SLR1)—a DELLA protein—providing further evidence of the interaction between JA and GA [67]. These findings confirm that antagonistic JA–GA crosstalk modulates plant growth and development under stress conditions. 

### 4.3. JA–SA Crosstalk

SA can activate the defense responses of plants against several plant pathogens in addition to its role in plant responses to salinity [68], light [69], cold [70], and other abiotic stresses [71]. Generally, the pathways of both hormones, JA and SA, are antagonistic, and this interaction induces plant resistance to various stresses [72]. Previous studies have examined the mechanism of JA–SA crosstalk, and have shown that several genes function in SA–JA antagonism, including *MYC2*, *PDF 1.2* (plant defensin 1.2), *TGAs* (TF family) [73], *MAPK* (mitogen-activated protein kinase) [74], *NPR1*, *ERF1*, *WRKY62*, *WRKY70*, *GRX480* (glutaredoxin 480), *ORA59* (octadecanoid-responsive *Arabidopsis* AP2/ERF 59), and *JAZs* [74]. The ortholog *NPR1* was identified in the ancestor of all land plants, suggesting that JA–SA crosstalk might occur in all plants [75]. Moreover, JA modulates the interaction between MYC2 and three NAC (TF family) genes (*ANAC019*, *ANAC055*, and *ANAC072*) to block the accumulation of SA, and these NAC TFs regulate the expression of genes involved in SA biosynthesis [76]. *MPK4* serves as a positive regulator of *GRX480* (SA signaling pathway) but negatively regulates MYC2 (JA signaling pathway) [16], and *GRX480* specifically binds to TGAs, which modulate the expression of *PR1*. Glutaredoxin (*GRX*) genes can block the expression of the JA response gene *ORA59* [77].

Interactions between JA and SA can protect plants against chilling injury, as demonstrated in pomegranate fruits [78]. These two hormones also improve plant tolerance to drought stress [79]. In *Arabidopsis*, JA treatment causes all genes and proteins involved in the oxidative, biotic, and abiotic stress responses to be overexpressed, while few are induced by SA treatment. Furthermore, negative crosstalk has been observed due to the combination of the two hormones; for example, SA exhibits negative control over the JA pathway [80]. This negative crosstalk has been reported in tomato plants after an attack from mealybugs, wherein the deficiency of SA leads to activation of JA and enhancement of the plant defense response [81]. Recently, a study investigated the contributions of JA and SA to ectomycorrhizae between the roots of grey poplar and the fungus *Laccaria bicolor*, and reported the regulation of gene clusters by their crosstalk [82]. 

### 4.4. JA–BR Crosstalk

Brassinosteroids (BRs) are considered the sixth class of plant hormones [83] that promote several developmental processes in plants, including cell division and growth, vascular differentiation, flowering, and leaf senescence [84], as well as modulation of gene expression [85]. In addition, BRs can induce disease resistance in tobacco (*Nicotiana tabacum*) and rice (*Oryza sativa*) [86]. The interaction between JA and BR has been examined in several studies. BR negatively regulates JA inhibition of root growth through modulation of the JA signaling pathway in *Arabidopsis* [87]. In the plant defense, BR antagonism with the JA pathway suppresses rice defense against root-knot nematodes [88]. Recently, a clear link between BR and JA signaling has been described, suggesting that OsGSK2 [glycogen synthase kinase 3 (GSK3)-like kinase, a key suppressor of BR signaling] enhances plant antiviral defenses by directly destabilizing OsJAZ4, thereby activating JA signaling [89]. These results show that the downregulation of BR biosynthesis generally causes an elevation of JA biosynthesis-related genes, suggesting negative crosstalk between JA and BR [64,88]. 

### 4.5. JA–ET Crosstalk

The interactions between jasmonate and ET demonstrate an interesting case of both synergism and antagonism. JA and ET are frequently described as playing coordinated roles in plant defense. JA/ET signaling is activated to regulate stress response when plants are infected by necrotrophic fungi and insects. It has been reported that these two hormones are both required for immune response and defense [90,91,92]. 

ET-insensitive 3 (EIN3) and its homolog EIN3-like 1 (EIL1)—TFs responsive to ET—are core components of the ET signaling pathway, while S2 is an important component of the JA signaling pathway. The interactions between JA-activated MYC2 and ET-stabilized EIN3/EIL1 regulate plant development and defense against insect attacks. *ERF1* (the downstream gene of EIN3/EIL1) expression is attenuated due to inhibition of EIN3/EIL1 by MYC2, ultimately causing suppression of plant resistance to necrotrophic fungi [90]. On the other hand, the interaction between EIN3/EIL1 and MYC2 can weaken the repression of MYC2 to defend against generalist herbivores, repressing the expression of the wound-responsive gene *VSP2* and herbivore-inducible gene *CYP79B3* through the JA signaling pathway [91,92]. These results suggest that JA and ET antagonistically regulate plant defense. 

However, the degradation of JAZ proteins is key to the synergistic effects of JA and ET. In the presence of increased JA, JAZs are degraded and therefore the interaction between HDA6 and EIN3/EIL1 is reduced and EIN3/EIL1 transcriptional activity is enhanced. In combination with ET activity stimulating EIN3/EIL1 protein accumulation, the key TFs EIN3 and EIL1 enhance the transcription of *ERF1* and *ORA59* and activate the ethylene response factor (ERF) branch of the JA pathway [93]. Consequently, the JA-responsive marker gene *PDF1*.2 is activated to support the defense against pathogens. Moreover, a new report demonstrates that the ET-induced transcriptional activators ERF15 and ERF16 can trigger a rapid JA burst in response to herbivore attack [94]. These interactions between JA and ET are associated with enhanced defenses against pathogens and herbivores [95].

### 4.6. JA–ABA Crosstalk

**Figure 5:**
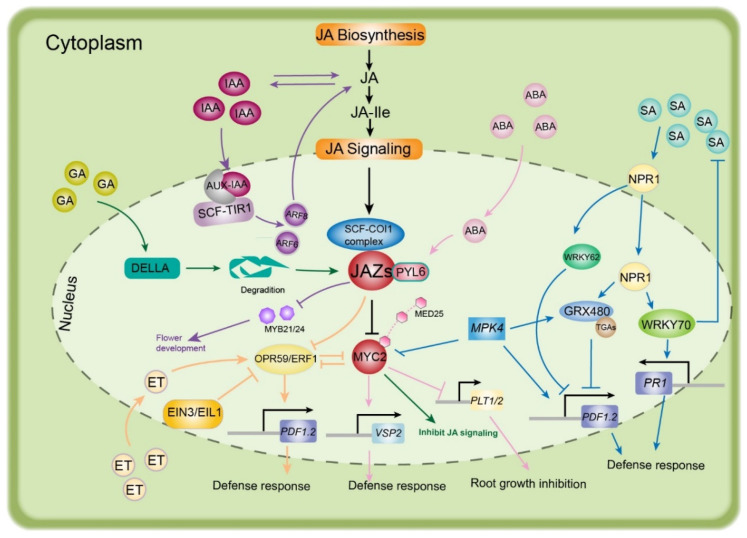
An integrated view of JA biosynthesis and signaling, including signaling interactions of JA with auxin, GA, SA, ET, and ABA in plant responses to stresses. See the text for further details. Lines of different colors represent the pathways of specific hormones. Abbreviations: SA, salicylic acid; NPR1, non-inducible PR1; GRX480, redox regulators glutathione; TGAs, TGACG-binding factors. PR1, pathogenesis-related protein 1; PDF1.2, plant defensin 1.2; MPK4, Mitogen-activated protein kinase 4; ABA, abscisic acid; PYL6, abscisic acid receptor; MED25, mediator 25; PLT1/2, plethora 1/2; VSP2, vegetative storage protein 2; ET, ethylene; OPR59, octadecanoid-responsive *Arabidopsis* AP2/ERF-domain protein 59; ERF1, ethylene response factor 1; EIN3, ethylene insensitive 3; EIL1, EIN3-like1; GA, gibberellic acid; IAA, indole acetic acid; AUX-IAA, auxin/Indole-3-acetic acid; SCF, [kinetochore protein 1 (SKP1)-CULLIN1 (CUL1)-F-box]; COI1, coronatine insensitive1; TIR1, transport inhibition response1; ARF6, auxin response factor 6; ARF8, auxin response factor 8; JA, jasmonic acid; JA-Ile, jasmonic acid isoleucine conjugate; JAZ, jasmonate ZIM domain.

The interaction between the ABA and JA signaling pathways is commonly involved in the responses to various biological stresses in plants [96,97]. In recent years, resistance to insects and necrotrophic fungal pathogens has been reported to be positively regulated by ABA and JA interactions [98,99]. Pyrabactin resistance 1-like proteins (PYLs) are a family of ABA receptors that regulate metabolic reprogramming via the JA signaling pathway in *A. thaliana* and tobacco [100,101]. Yeast two-hybrid assays showed that the direct interaction of PYL6 and MYC2 is strongly enhanced in the presence of ABA, which promotes the expression of JAZ8 and decreases JAZ6 activity. Thus, PYL6 has been identified as a transcriptional modulator through its interaction with MYC2 [102]. Recently, silenced *VvPYL4* was found to repress the transcriptional activities of MYC2, JAZ, and JAR1, which are associated with the defensive response to downy mildew in grapevine [97]. Hence, PYL and JAZ-MYC2 play critical roles in JA–ABA crosstalk to regulate plant defense responses. The interaction between MYC2 and MED25 can enhance the expressional activity of the *VSP2* gene (JA-responsive gene) to resist herbivorous insect attacks (Figure 5). 

## 5. JA in Abiotic Stress Tolerance

JA is extensively involved in plant responses to environmental cues, including abiotic stresses. Under abiotic stress, the role of JA is in the physiological and molecular responses to drought, cold, salt, heavy metals, heat, and other stressors. In this section, we discuss the functions and reactions of JA in plant resistance to abiotic stresses.

### 5.1. Drought

**Figure 6:**
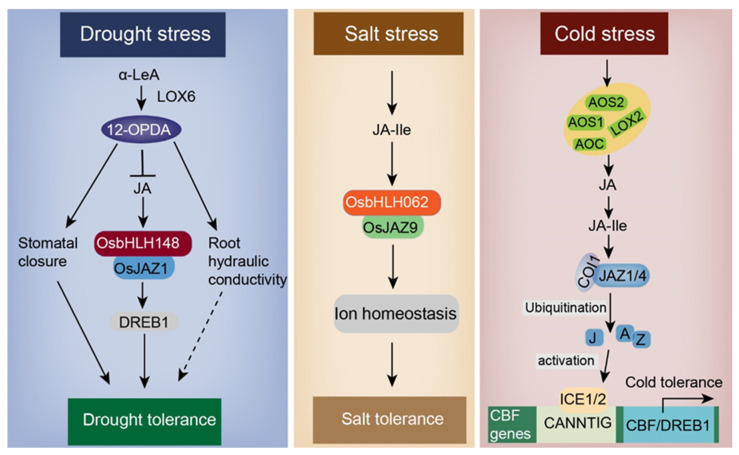
Jasmonic acid (JA)-mediated drought, salt, and cold stress responses in plants. Under drought, salt, and cold stresses, the JA signaling pathway can be activated. When Oryza sativa encounters drought, expression of OsDREB1 is induced. The interaction of OsbHLH148 with OsJAZ1, along with JA-mediated root hydraulic conductivity and stomatal closure, improves drought tolerance in rice. Induced by the stimulus of salt stress, OsbHLH062 interacts with OsJAZ9, resulting in ion homeostasis and improved salt tolerance. In *A. thaliana*, cold stress induces the expression of AOS1, AOS2, AOC, and LOX2, which cause JA-Ile epimerization from JA. Furthermore, JA-Ile facilitates the interaction of JAZ with the F-box protein COI1 through ubiquitination to regulate the expression of downstream genes, thereby achieving cold tolerance. Abbreviations: 12-OPDA, 12-oxophytodienoic acid; JA, jasmonic acid; bHLH148, basic helix-loop-helix 148; JAZ, jasmonate zim domain protein; DREB, dehydration-responsive-element-binding; LOX6, 13-lipxoxygenase 6; JA-Ile, jasmonic acid isoleucine; AOS, allene oxide synthase; AOC, allene oxide cyclase; CBF, C repeat binding factor; ICE, inducer of CBF expression; COI1, coronatine insensitive 1. (Redrawn based on [101]).

Water scarcity is likely the most serious and prevalent abiotic stressor affecting plant growth and development, as well as crop quality and productivity. Stomatal opening controls CO_2_ and H_2_O exchange between the plant and the environment [103]. Thus, one of the main stress adaptation strategies to improve drought resistance is control of stomatal transpiration (stomatal closure) in plants [104,105]. Elevated levels of OPDA are associated with decreased stomatal aperture and enhanced drought tolerance in *Arabidopsis* [106]. Drought stress impedes the conversion of OPDA to JA; thus, accumulated OPDA can induce stomatal closure as a drought-responsive regulator [107]. Previous studies indicated that *LOX* genes are essential to the synthesis of 12-OPDA and play a key role in drought tolerance. Under drought stress, increased 12-OPDA induced by LOX6 improved the plant response, mainly through promoting stomatal closure in the absence of ABA (Figure 6) [108]. In addition, the basic helix-loop-helix (bHLH) protein in rice (*OsbHLH148*) is involved in drought resistance, interacting with OsJAZ1 to cause activation of an important TF, OsDREB1, to support drought stress tolerance. JAZ1 interacts with the putative OsCOI1 only in the presence of coronatine. These results suggest that *OsbHLH148* functions as an initial response of jasmonate-regulated gene expression, activating the OsbHLH148–OsJAZ–OsCOI1 signaling module in rice in response to drought tolerance [109]. 

A growing number of studies on various plants indicate the involvement of JA synthesis genes in drought resistance. For example, ectopic overexpression of *CMLOX10* from *Cucumis melo* var. *makuwa* enhanced drought tolerance and decreased stomatal aperture in *Arabidopsis*, whereas the JA application induced stomatal closure and alleviated drought stress in *C. melo* var. *makuwa* [110]. Previous studies have shown that the regulatory module of *RhHB1/RhLOX4* could mediate a JA feedback loop, enhancing dehydration tolerance by fine-tuning bioactive JA levels in dehydrated rose flowers (*Rosa hybrida*) [111]. Some NAC TF family members such as *VvNAC17* and *VvNAC26* in *Vitis amurensis*, have also been shown to regulate endogenous JA synthesis and upregulate other stress-responsive genes, thereby playing a positive role in drought tolerance [112,113]. Recently, in the JA signaling pathway, the involvement of JAZ in drought tolerance has been reported. Overexpression of OsJAZ9 reduced leaf width and stomatal density, thereby lowering the leaf transpiration rate and improving rice tolerance to water deficit stress [114]. The application of exogenous JAs has also improved drought resistance in wheat (*Triticum sativum*) [115], strawberry (*Fragaria* × *ananassa* Duch.) [116], rice (*Oryza sativa*) [117], pearl millet (*Pennisetum glaucum* L.) [118], and sugar beet (*Beta vulgaris* L.) [119]. The contents of total soluble proteins, proline (Pro) and malondialdehyde (MDA), and the activities of antioxidant enzymes (including superoxide dismutase, peroxidase, and catalase) all increased notably by the application of exogenous JAs, enhancing the drought tolerance of plants [115,120].

### 5.2. Cold Stress

Cold stress adversely affects plant growth and development. In general, cold stress can be divided into low-temperature stress and freezing damage [121]. Low-temperature (chill) stress generally occurs at 0–15 °C and causes cell dysfunction, whereas freezing stress occurs below 0 °C due to the production of intracellular ice crystals, which cause mechanical damage [122]. To alleviate the damage caused by cold stress, plants have evolved complex mechanisms for regulating stress-related gene expression. Over the past two decades, C-repeat binding factor/dehydration-responsive-element-binding protein 1 (*CBF/DREB1*)-dependent cold signaling has been widely investigated. The signaling pathway of ICE–CBF transcriptional regulation plays a central role in maintaining plant development and survival under cold-stress conditions [123]. In *A. thaliana*, ICE1 and ICE2, two bHLH TFs, positively regulate the expression of *CBFs* through direct binding to CANNTG cis-elements present in the *CBF* promoters [122,123]. Under normal conditions, the transcriptional activities of ICE–CBF pathway-related genes are inhibited by the complex of JAZ1/4 and ICE1/2. In rice, several JA biosynthesis-related genes (including *AOC*, *AOS1*, *AOS2*, and *LOX2*) and signaling genes (*COI1a* and *bHLH148*) positively regulate the cellular response to cold stress [60,124]. The COI1–JAZ co-receptor perceives JA-Ile, promoting the interaction of JAZ with COI1 and resulting in the degradation (ubiquitination) of JAZs through the 26S proteasome [101]. Consequently, the ICE–CBF pathway is activated and induces the expression of certain cold-related genes to enhance the capacity for cold tolerance (Figure 6).

Jasmonates may act as upstream signals in the ICE–CBF/DREB1 pathway to positively regulate freeze tolerance in apples (*Malus* × *domestica*). Although *MdMYC2* was shown to increase the expression levels of *MdCBF1*, *MdCBF2*, and *MdCBF3*, resulting in increased freezing tolerance, the overexpression of *MdJAZ1* or *MdJAZ4* offset the promotive effect of *MdMYC2* on cold tolerance [125]. The novel B-box (BBX) protein MdBBX37 positively regulates JA-mediated cold-stress resistance, whereas MdJAZ1/JAZ2 interacts with MdBBX37 to negatively regulate JA-mediated cold tolerance [126]. In addition, the ICE-like transcription factors HbICE1/ICE2 have been shown to mediate jasmonate-regulated cold tolerance in the rubber tree (*Hevea brasiliensis*) [127].

Low temperature can induce the production of many secondary metabolites and upregulate JA-related genes. For example, cold stress promotes artemisinin biosynthesis due to increased endogenous JA in *Artemisia annua* [128]. Under cold stress, PtrMYC2 can bind to the promoter of betaine aldehyde dehydrogenase (BADH)-like gene *PtrBADH-l* and activate its expression to promote glycine betaine synthesis in *Poncirus trifoliata*. It also explains why plants have cold tolerance from another perspective [129]. 

MeJA treatment also promotes cold tolerance in plants. JA synthesis-related genes were activated and the endogenous JA content increased with MeJA treatment, leading to markedly higher contents of antioxidant metabolites (glutamic acid, sucrose, and galactose) in pepper fruits, which suggests that MeJA could effectively alleviate the damage caused by low temperature [130]. Similarly, MeJA treatment prior to harvest not only effectively increased the antioxidant content of lemon fruits without affecting yield or quality but also improved the tolerance of lemons to postharvest chilling injury and decay [131]. These results reveal that JA treatment can minimize the injury caused by low-temperature stress through stimulation of defensive compound production by antioxidant systems.

### 5.3. Salt Stress

Salt stress has become a severe problem on a worldwide scale, especially in arid and semi-arid regions [132]. To adapt to abnormally high salinity in the soil, plants tend to evolve complex mechanisms for regulating molecular, physiological, and biochemical processes. Salt stress can trigger activation of the JA signaling pathway and induce JA biosynthesis in plants. For example, in *Arabidopsis* roots, salt stress activated the JA signaling pathway, followed by cell elongation inhibition, in the elongation zone [133]. Similarly, in *Dendrobium officinale* leaves, JA may act as a signal molecule, inducing JA biosynthesis to contribute to plant adaptation to salt stress and promote flavonoid biosynthesis [134]. In rice, major oxidative damage under salt stress was caused by excessive reactive oxygen species (ROS), whereas enhanced ROS-scavenging activity in an OPDA-deficient mutant was linked to JA biosynthesis [135]. Thus, the JA signaling and biosynthesis pathways play important roles in plant salt stress responses.

Some studies have shown that salt tolerance may be regulated by JA-related genes. For example, LOX3, an enzyme involved in JA synthesis, was shown to be increasingly induced under salt stress and enhanced salt tolerance in *Arabidopsis* [136]. *HKT1*, an Na^+^ transporter, is a negative regulator of lateral root development under salt stress. However, the JA-Ile transporter, glucosinolate transporter1 (GTR1/NPF2.10) alleviates the repression of lateral root development due to salt stress by mediating JA signaling and repressing *HKT1* expression [137]. In *Arabidopsis*, the important role of *MYC2* in the salt response is mediated by the JA signaling pathway [133]. In addition, rice OsbHLH062 interacts with OsJAZ9 to regulate ion homeostasis under salt stress, thereby improving salt tolerance [138] (Figure 6). The overexpression of *GaJAZ1* significantly improves salt tolerance by reprogramming the expression of defense-related genes in *Gossypium hirsutum* [139].

Many studies have examined salt tolerance improvement through exogenous JA application [132,133,134]. For example, the MeJA application improved tolerance to high-salt stress in the recretohalophyte *Limonium bicolor* [140]. JA effectively protected wheat seedlings from salt stress damage by enhancing antioxidant enzyme activity and antioxidative compound concentrations [141]. Foliar JA application enhanced salinity stress tolerance in soybean (*Glycine max* L.) seedlings under salt stress, possibly through the regulation of auxin signaling and stomatal closure [142]. However, the combination of salinity and MeJA intensified plant growth inhibition and senescence [143]. Exogenous MeJA amplified salt stress-induced growth inhibition and prioritized defensive responses (e.g., antioxidant defense, osmotic adjustment, and ion homeostasis) in *Nitraria tangutorum* [144].

JA mainly interacts with ABA signaling pathways to regulate salt stress tolerance. For example, the combined application of ABA and JA activated the salt stress-protective mechanism of strawberry plants [145]. In *Pohlia nutans*, overexpression of *PnJAZ1* increased tolerance to salt stress, and ABA treatment via PnJAZ1 mediated JA-ABA synergistic crosstalk [146].

### 5.4. Heat Stress

The threat of heat stress to plant production is a global issue. A series of physiological and ecological changes occur in plants due to high temperatures, affecting their growth and development [147]. Heat stress can result in a serious decline in agricultural economic yield due to adverse effects on crop physiology, including cell membrane damage, enzyme inactivation, inhibition of photosynthesis, and enhanced respiration [148,149]. 

JA application has been found to improve the heat-shock tolerance of plants. Exogenous MeJA significantly improved the heat tolerance of perennial ryegrass by altering osmotic adjustment, antioxidant defense, and JA-responsive gene expression [150]. High temperature-induced tomato stigma exsertion, causing fruit set failure, whereas exogenous JA application was more effective than auxin treatment in rescuing tomato stigma exsertion through regulation of the JA/COI1 signaling pathway [151].

A severe problem affecting the process of seed production of photo-thermo-sensitive genic male sterile rice is that the low spikelet opening rate leads to reduced grain yield at high temperature, along with significantly lower contents of JA and MeJA in rice leaves relative to control [152]. On the other hand, the application of JA or MeJA significantly increases the concentration of JAs in rice leaves under high-temperature stress. Meanwhile, the antioxidant system and osmotic adjustment capacity of leaves were markedly enhanced, which improved the spikelet flowering rate and reduced the restriction of spikelet opening caused by high temperature. Nevertheless, JA notably inhibits stomatal development and induces stomatal closure [153]; therefore, JA is not suitable for use in rice, which maintains thermotolerance through leaf cooling via transpiration.

Generally, heat-shock proteins alleviate heat-induced damage through the plant heat response pathway. Numerous studies have shown that exogenous JA applied to plants before heat tolerance can reduce heat-induced damage, suggesting that JA plays an important role in plant thermotolerance [154]. However, the mechanisms underlying heat response pathway interactions with JA are rarely explored [155]. Several studies have suggested that JA might regulate plant heat stress responses through a subset of the WRKY superfamily that is induced by JA [149,156,157].

### 5.5. Heavy Metal Toxicity

Heavy metals originating from industrial waste materials or the natural environment, including lead (Pb), mercury (Hg), copper (Cu), and cadmium (Cd), have toxic effects on plant growth and development when their levels in the plant body exceed a threshold [158].

JA is widely involved in plant responses to heavy metals, but the underlying molecular mechanism remains unclear. A recent report indicated that JA synthesis gene expression was rapidly regulated in *A. thaliana* under Cd treatment, with endogenous JA concentrations increasing rapidly following Cd stress in the roots. Exogenous application of MeJA not only significantly reduced the concentration of Cd in root and shoot tissues but also inhibited the expression of the *AtIRT1* (iron-regulated transporter), *AtHMA2* (heavy metal ATPase), and *AtHMA4* (heavy metal ATPase) genes. In other words, JAs can reduce the expression of genes that facilitate Cd uptake and long-distance translocation, thereby reducing the Cd concentration in root cell sap and relieving Cd stress [159]. Several studies have shown that exogenous JA can mitigate the detrimental effects of heavy metals on plants by regulating the activity of the antioxidant defense system, increasing chlorophyll content, and inducing secondary metabolites. For example, JA application facilitated the alleviation of negative Ni effects in soybean seedlings through the enhanced activity of osmolytes, which are antioxidant enzymes [160]. Exogenous JA application in wheat resulted in a protective effect against Cu stress and significantly increased glutathione S-transferase (*GST*) gene expression, related to ROS scavenging [161]. Under Cu stress across a range of concentrations, JA treatment could efficiently relieve heavy metal-induced inhibition of alfalfa growth by increasing leaf chlorophyll content, antioxidant enzyme activity, MDA, and hydrogen peroxide [162]. Similarly, the addition of exogenous JA increased secondary metabolites including total phenols, polyphenols, flavonoids, anthocyanin, organic acids, and metal-chelating compounds in the seedlings of Pb-contaminated tomato, thereby reducing the toxicity of Pb [163]. Combined JA, SA, and proline treatment eliminated negative effects on maize growth and development caused by Pb stress [164].

## 6. JA in Biotic Stress Tolerance

Many studies have demonstrated that JA plays an important role in plant biotic stress responses, mainly to pathogens or insects. In this section, we discuss recent findings on the involvement of JA in plant resistance to various types of biotic stress.

### 6.1. Insect Resistance

Plant defense responses to insects are regulated by molecular signals, among which the most important signaling molecule is JA [23,165]. Plants are frequently damaged by insects, many of which are herbivores, eliciting a rapid increase in endogenous JA levels [166]. Interestingly, when plants are sprayed with JA, plant secondary metabolites and systemic resistance are induced to avoid the damage by insects, although JA itself is non-toxic to insects [167,168].

Herbivore attack always induces accumulation of defensive proteins in plants, which affect the digestive physiology of pests and thereby protect plants from herbivory [169]. These defensive proteins could be induced by JAs. For example, the important defensive enzyme polyphenol oxidase (PPO) can be induced by exogenous JA or MeJA, and can effectively protect plants from herbivores [170]. In maize, MeJA enhanced toxic protein production and induced plant defense mechanisms to *Ostrinia furnacalis* [171]. In addition, many secondary metabolites induced by jasmonates, which are products of complex branching metabolic pathways, play active roles in the insect resistance of plants. Over the past decade, secondary metabolites have been identified that induce resistance to the attacks of herbivorous insects, mainly including tannins, total phenols, total flavonoids, and lignin [172,173].

**Figure 7:**
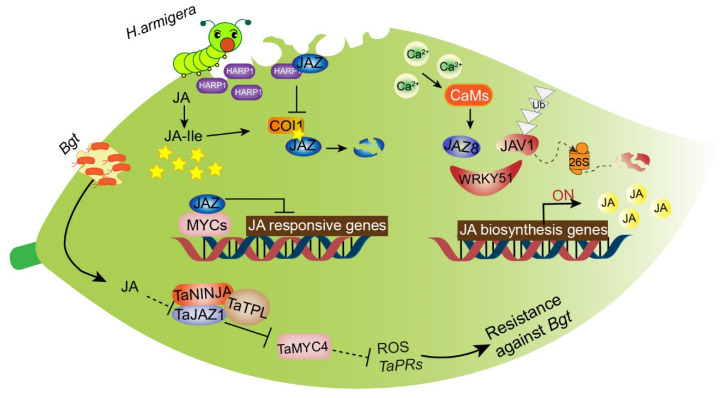
The JA signaling pathway in response to biotic stresses in plants. In wheat, pathogen and insect damage activate the JA signaling pathway. When bread wheat is inoculated with *Bgt*, JA accumulation results in TaJAZ1 degradation to release TaMYC4, inhibiting reactive oxygen species accumulation and *TaPRs* expression and thereby showing resistance to *Bgt*. On the other hand, the chewing insect-derived effector HARP1, secreted from *Helicoverpa armigera*, accumulates in leaves and then interacts with JAZ to repress the interaction of COI1 and JAZ, thus preventing JAZ degradation and inactivating the expression of JA-responsive genes to decrease the plant’s resistance to insect damage. In addition, during insect herbivory, the JJW complex is disintegrated due to the rapid induction of Ca^2+^/calmodulin-dependent phosphorylation of JAV1, thereby de-repressing JA biosynthesis genes and causing a rapid burst of JA for plant defense. Abbreviations: HARP1, caterpillar-derived effector; ROS, reactive oxygen species; PR1/2, pathogenesis-related gene1/2; JAV1, Jasmonate-associated VQ domain protein 1; JA, jasmonic acid; JAZ, jasmonate zim domain protein; JA-Ile, jasmonic acid isoleucine; NINJA, novel interactor of JAZ; TPL, topless.

To deter or poison herbivores or attract their natural enemies, plants have evolved specific and complex mechanisms that are regulated through phytohormone signaling, especially the JA signaling pathway [174]. Although JAs are widely accepted to play a critical role in inducing plant defenses against herbivorous insects, the process through which plants initially activate JA biosynthesis during herbivore encounters remains unclear. In healthy plants, *jasmonate-associated VQ* domain protein 1 (JAV1) forms a novel complex with JAZ8 and WRKY51, known as JAV1-JAZ8-WRKY51 (JJW), which inhibits the expression of JA biosynthesis genes. Thus, JA can maintain a low basal level to support normal plant growth. However, when plants are injured due to insect attacks, the calmodulin-dependent phosphorylation of JAV1 is activated in response to calcium influx. The JJW complex is degraded and JA biosynthesis is activated, causing the content of JA to increase rapidly for plant defense [175]. *OsAOS1* and *OsAOS2* are both involved in herbivore-induced JA biosynthesis and play a vital role in determining rice resistance to herbivore consumption [176]. Tomato *TomLoxD* overexpression leads to elevated wound-induced JA biosynthesis, enhancing resistance to insect herbivory attack [177]. *CsOPR3*, a JA biosynthesis gene in tea plants, plays an important role in defense against herbivorous insects [178]. *OsCOI1* is a signaling component that controls JA-regulated defense against chewing insects in rice plants via the induction of trypsin protease inhibitor (TrypPI), polyphenol oxidase (PPO), and peroxidase (POD) [179]. MYC2, MYC3, and MYC4 are major regulators of *Arabidopsis* resistance to a generalist herbivore [180]. However, recently, JAZ proteins were reported to act as targets for interference with immune signaling by insect effectors. HARP1, a chewing insect-derived effector obtained from oral secretions of *Helicoverpa armigera*, interacts directly with JAZ to repress JAZ protein degradation (Figure 7). Thus, JA signal transduction is blocked and the immune response is inhibited [181]. HARP1 can thereby reduce wound-induced defense signaling and increase plant susceptibility to insect damage.

### 6.2. Plant Disease Resistance 

JAs, endogenous signaling molecules, are not only involved in the insect resistance mechanism of plants but also has a clear defensive effect on necrotrophic pathogens [165].

Previous studies have shown that the exogenous application of JA or MeJA can induce the expression of plant defense genes. Similarly, with JAs treatment, the expression of several genes in the JA signaling pathway was induced significantly, thereby improving resistance to necrotrophic pathogens [182]. MeJA treatment induced significant upregulation of *PR4*, *PR5*, and *PEROXIDASE (PEROX)* to defend against *Fusarium graminearum* in wheat [183]. Significant changes in defensive enzymes and secondary metabolites occur, which play important roles in plant resistance against pathogen invasion [184,185]. Furthermore, the activities of peroxidase and PPO, which can effectively inhibit the incidence of green and blue molds, increased markedly in citrus fruit with exogenous JA treatment [184]. In wheat infected by *Fusarium culmorum*, application of MeJA can significantly decrease the level of H_2_O_2_ contents and lipid peroxidation [186]. After MeJA treatment, a large number of genes were significantly upregulated, including genes related to terpenoid biosynthesis, phenylalanine metabolism, plant-pathogen interaction, JA biosynthesis, and the JA signaling pathway, which improved the resistance of *Panax notoginseng* to *Fusarium solani* during growth [187].

In recent years, the molecular mechanisms of processes involving JA and its derivatives in plant disease resistance have been widely studied. OPDA, as an important intermediate in the JA biosynthesis pathway, plays a role in plant defense against *Botrytis cinerea* by potentiating callose deposition [188]. CATALASE2 (CAT2) promotes JA-biosynthetic acyl-CoA oxidase (ACX) activity to enhance plant resistance to the necrotrophic *Botrytis cinerea* [189]. In addition, prior exposure of *Arabidopsis* seedlings to mechanical stress enhanced JA-mediated defense against necrotrophic pathogens [190], and GhCOI1 is an essential signaling component that controls the JA-regulated defense response against *Alternaria brassicicola* in *Gladiolus* plants [191].

Bread wheat (*Triticum aestivum* L.) often suffers from powdery mildew, caused by the biotrophic fungal pathogen *Blumeria graminis* f. sp. *tritici* (*Bgt*), whereas transgenic bread wheat lines overexpressing the TaJAZ1 fragment lacking the Jas motif exhibited enhanced expression of the pathogenesis-related genes *TaPR1/2* to protect against *Bgt*. Meanwhile, a co-repressor complex comprised of TaJAZ1, TaNINJA, and TaTPL was found to inhibit the transcriptional activity of TaMYC4 as a result of the degradation of TaJAZ1 [192]. Overexpression of the JA-related *OsbHLH034* gene in rice induced the JA-hypersensitive phenotype and increased resistance to rice bacterial blight [193]. GhJAZ2 interacted with GhbHLH171 to restrain the JA-mediated defense response to the fungus *Verticillium dahliae* in cotton [194]. JAZ8 repressed the transcriptional function of WRKY75, whereas *JAZ8* overexpression repressed plant defense responses to necrotrophic pathogens in *Arabidopsis* [195]. These results demonstrate that resistance against necrotrophic pathogens can be strengthened by JA signaling-related genes (Figure 7).

To sum up, both endogenous and exogenous JAs play an important role in plant responses to abiotic stress and biotic stress. The protective roles of JAs in response to drought, cold, salt, heat heavy, insect and disease stresses are listed in Table 1.

## 7. Conclusions and Future Directions

As the key hormones mediating biotic and abiotic stress responses, JAs have been the focus of extensive and intensive research related to stress-induced regulation of plant growth and defense. Over the years, JA synthesis, signaling, and metabolism pathways have gradually been outlined using model plants (e.g., *Arabidopsis* and rice), providing important references for other plants. However, few studies of certain important agricultural crops and economic trees have been conducted, although biotic and abiotic stresses have led to major negative economic impacts on the production of these plants. The applications of JA in agricultural production to date are limited. Therefore, further studies of JAs in important economic plants are essential basic research needed to facilitate practical application against plant stressors. Generally, JAs are beneficial to enhance defensive resistance, or stress tolerance, and therefore have broad application potential in agriculture and forestry as anti-stress protective agents. However, the side-effects of MeJA application, such as inducing seedling mortality and reduction height growth, should not be neglected [197,198].

The crosstalk of JA with other hormones plays a vital role in the regulation of plant growth and development under stress conditions. However, research on the identification and characterization of JA functions and interactions with other hormones remains inadequate. At present, most studies on JA crosstalk have focused on specific stages of plant development, and the dynamics of hormonal interactions across the plant lifespan remain elusive. In addition, research on the responses of JAs to combinations of multiple environmental signals is scarce, despite the common co-occurrence of multiple environmental stresses in nature.

Previous studies of JAs have generally focused on JA functional mechanisms within plant cells, while the tissue, organ, individual, and inter-individual levels remain unclear. As JAs, and especially OPDA and its derivatives, are mobile and can be transported to different parts of plants to function in plant defense systems, future investigations of JA transmission and its applications are needed. In particular, MeJA is widely found among the leaf and floral volatile components of various plants [199]. Therefore, the function of MeJA transmission via air is worthy of further research to expand the range of plant defenses available for improving agricultural production.

Among plant natural products, JAs are crucial for the plant defense system. JA biosynthesis is dependent on the environmental conditions under which the plant is grown. Molecular biology techniques such as gene editing can be used to modify the genetic regulation of JA synthesis to create stress-resistant crop cultivars. Furthermore, exogenous JAs are widely used as anti-stress protective agents by enhancing plant resistance. The further advancement of nano-biotechnology will facilitate the development of nanocarriers for the rapid and efficient delivery of exogenous JAs to targeted plant cells. 

## Figures and Tables

**Table 1:** Regulation mechanism of endogenous and exogenous JAs in response to abiotic and biotic stresses.

Type	Stress	Plant Species	JAs	Protective Role	Reference
Abiotic stress	Drought	*Oryza sativa*	Endogenous	OsbHLH148 was interacted with OsJAZ1 and COI1 to activate DREB1 constituted OsbHLH148–OsJAZ–OsCOI1 signaling module	[107]
	Drought	*Oryza sativa*	Endogenous	Overexpression of OsJAZ9 reduced leaf width and stomatal density	[114]
	Drought	*Triticum sativum*	Exogenous (JA)	Increased the accumulation of some osmoregulation compounds and regulated the activity of antioxidant enzymes as well as morphological modulation attained by the restoration of shoot/root ratio	[115]
	Drought	*Pennisetum glaucum*	Exogenous (JA)	Improved chlorophyll, relative water content and activities of antioxidative enzymes	[118]
	Cold	*Oryza sativa*	Exogenous (JA)	Induced several JA-related genes (including AOC, AOS1, AOS2, LOX2, COI1a, and bHLH148 positively	[60,124]
	Cold	*Malus × domestica*	Endogenous	Overexpression of *MdMYC2* increased the expression levels of *MdCIbHLH1*, *MdCBF1*, *MdCBF2*, and *MdCBF3*.	[125]
	Cold	*Malus × domestica*	Endogenous	MdJAZ1/JAZ2 interacted with MdBBX37 to negatively regulate JA-mediated cold tolerance.	[126]
	Cold	*Poncirus trifoliata*	Endogenous	PtrMYC2 can bind to the promoter of *PtrBADH-l* and activate its expression to promote glycine betaine synthesis to enhance cold tolerance	[129]
	Cold	*Capsicum annuum*	Exogenous (MeJA)	Activated JA synthesis-related genes, increased endogenous JA content and contents of antioxidant metabolites (glutamic acid, sucrose, and galactose)	[130]
	Salt	*Oryza sativa*	Endogenous	OsbHLH062 interacted with OsJAZ9 to regulate ion homeostasis	[138]
	Salt	*Gossypium hirsutum*	Endogenous	Overexpression of *GaJAZ1* significantly improves salt tolerance by reprogramming the Expression of defense-related genes	[139]
	Salt	*Triticum aestivum*	Exogenous (JA)	Enhanced activities of antioxidant enzymes and accumulated antioxidative compounds	[141]
	Salt	*Glycine max*	Exogenous (JA)	Regulated the interaction between plant hormones and hydrogen peroxide	[142]
	Heat	*Oryza sativa*	Exogenous (MeJA)	Increased the concentration of JAs; enhanced the antioxidant system and osmotic adjustment capacity of leaves; and improved the spikelet flowering	[153]
	Heat	*Solanum lycopersicum*	Exogenous (JA)	Rescued tomato stigma exsertion via regulating the JA/COI1 signaling pathway	[151]
	Heat	*Solanum lycopersicum*	Endogenous	Overexpression of *TomloxD* enhanced the heat tolerance	[196]
	Heavy metal (Ni)	*Glycine max*	Exogenous (JA)	Enhanced osmolytes, activity of antioxidant enzymes and gene expression.	[160]
	Heavy metal (Cu)	*Triticum sativum*	Exogenous (JA)	Increased transcripts of the glutathione S-transferase (GST) gene	[161]
	Heavy metal (Pb)	*Lycopersicon esculentum*	Exogenous (JA)	Increased total phenols, polyphenols, flavonoids, anthocyanin, organic acids, and metal-chelating compounds in seedlings	[163]
Biotic stress	*Ostrinia furnacalis*	*Zea mays*	Exogenous (MeJA)	Induced plant defense mechanisms and enhanced toxic protein production	[171]
	*Chilo suppressalis* and *Niaparvata lugens*	*Oryza sativa*	Endogenous	OsAOS1 and OsAOS2 are both involved in herbivore-induced JA biosynthesis	[176]
	*Cnaphalocrocis medinalis* and *Nilaparvata lugens*	*Oryza sativa*	Endogenous	COI1 is also required for induction of trypsin protease inhibitor (TrypPI), POD and PPO	[179]
	*Sclerotinia sclerotiorum*.	*Brassica napus*	Exogenous (JA)	Increased the expression of several JA-related signaling genes to improve the resistance. Including *BnLOX2, BnAOS*, and *BnPDF1.2*	[182]
	*Fusarium culmorum*,	*Triticum aestivum*	Exogenous (MeJA	Significantly decreased the level of H_2_O_2_ contents and lipid peroxidation	[190]
	*Blumeria graminis* f. sp. *tritici* (*Bgt*)	*Triticum aestivum*	Endogenous	Overexpressed the *TaJAZ1* enhanced expression of the pathogenesis-related genes *TaPR1/2* to protect against *Bgt*	[192]
	*Verticillium dahliae*	*Gossypium hirsutum*	Endogenous	GhJAZ2 inhibited GhbHLH171 transcriptional activity to restrain the JA-mediated defense response to fungus	[194]
